# Mental Health Polypharmacy in “Non-Coded” Primary Care Patients: The Effect of Deprescribing

**DOI:** 10.3390/jcm13040958

**Published:** 2024-02-07

**Authors:** Waseem Jerjes, Daniele Ramsay, Harvey Stevenson, Karima Lalji

**Affiliations:** 1 Research and Development Unit, Hammersmith and Fulham Primary Care Network, Richford Street, London W6 7HY, UK; karima.lalji@nhs.net; 2 Faculty of Medicine, Imperial College London, London SW7 2DD, UK; daniele.ramsay18@imperial.ac.uk (D.R.); harvey.stevenson18@imperial.ac.uk (H.S.)

**Keywords:** mental health, polypharmacy, primary care, general practitioner

## Abstract

**Background**: Mental health (MH) polypharmacy, defined as prescribing multiple mental health medications for the same condition, presents significant challenges in clinical practice. With varying prevalence rates and an increasing trend, particularly in the UK, this deprescribing prospective quality improvement project aimed to address the complexities and risks associated with MH polypharmacy. **Patients and Methods**: A large primary care centre in London was selected for this project. Electronic records of 667 patients (non-coded in mental health lists) were analysed as a result of the absence of a Systematised Nomenclature of Medicine Clinical Terms (SNOMED CT) for mental health. Seventy-two non-coded patients exhibiting “same-class” as well as “adjunctive” and “augmentation” polypharmacy were identified. Their demographic and health data, including MH diagnoses, physical status, and lifestyle habits, were evaluated. This deprescribing prospective project included 68 patients and employed a model inspired by the Plan–Do–Study–Act (PDSA) cycle, focusing on reducing psychotropic, adjunctive, and augmentative medications while monitoring mental health control through face-to-face consultations using the Patient Health Questionnaire-9 (PHQ-9) and Generalised Anxiety Disorder Assessment-7 (GAD-7) scores, alongside physical health parameters. **Results**: The project revealed a significant decrease in the average number of psychotropic and adjunct medications from initial consultations to the end of the 18-month period. Additionally, a marked reduction in reported side effects and drug interactions was observed. Improvements in mental health control, as evidenced by PHQ-9 and GAD-7 scores, were noted. Physical health parameters, including BMI, blood pressure, heart rate, HbA1c, and cholesterol levels, also showed significant improvements. Educational initiatives for patients and clinicians were successfully implemented, contributing to these positive outcomes. **Discussion**: The project faced challenges like balancing medication reduction with mental health stability, patient apprehension, and the absence of standardised protocols. However, the successful reduction in medication numbers and the improvement in health outcomes highlight the effectiveness of the model. This project underscores the necessity of a tailored approach to MH polypharmacy, emphasising continuous education, clinical titration, and adherence to guidelines. Future research is needed to develop clear guidelines for medication combination in mental health care and to understand the long-term effects of polypharmacy in mental health populations. **Conclusions**: This project demonstrates the potential for significant improvements in the management of MH polypharmacy. By carefully managing medication reductions and employing a comprehensive care approach, including patient education and clinician training, the project achieved improvements in both mental and physical health outcomes. These findings suggest a promising direction for future practices in MH polypharmacy management.

## 1. Introduction

In the field of mental health (MH), physicians face challenges in managing complex cases, with some conditions posing life-threatening risks or showing resistance to treatment [1]. Polypharmacy has been a common approach in such difficult scenarios. Traditionally, this refers to prescribing more than one psychotropic medication to a patient, especially for treating the same condition, regardless of whether the medications belong to the same chemical class or share pharmacological actions [2]. However, the practice of polypharmacy, including its definition being based on the mere quantity of medications without considering clinical relevance or the necessity of the treatment regimen, is now being critically evaluated for its appropriateness in patient care [3]. The exact number of MH medications constituting polypharmacy remains undefined in the literature [1].

The National Association of State Mental Health Programme Directors (NASMHPD) has outlined various categories for addressing the growing prevalence and intricacy of mental health polypharmacy [4]. Firstly, “same-class” polypharmacy is characterised by the simultaneous use of multiple medications from the same pharmacological class. In contrast, “multi-class” polypharmacy involves the use of full therapeutic doses of medications from different classes to address the same symptom cluster. On the other hand, “adjunctive” polypharmacy is defined as the use of one medication specifically to mitigate the side effects caused by another medication from a different class. Moreover, “augmentation” polypharmacy is a strategy where one medication is used in lower-than-usual doses alongside another medication from a different class in full therapeutic doses to treat the same symptom cluster. These classifications provide a framework for understanding the various approaches and methodologies employed in mental health polypharmacy, reflecting its diverse and complex nature in clinical practice [5,6,7].

While reported overall prevalence rates of polypharmacy in mental health vary between 13 and 90%, there has been a clear overall trend towards polypharmacy and decline in mental health patients being treated with monotherapy [3]. One cross-sectional survey found polypharmacy to be more prevalent in men than women, peaking in those aged 25–45. However, mental health polypharmacy is increasing in geriatric and even adolescent populations, too [1].

The recent trend in mental health polypharmacy has seen a global increase, with the UK experiencing a notably significant rise. The NHS Business Services Authority (NHSBSA) in England has published its annual data report, which provides insights into mental health prescribing patterns over the past year [8]. This report highlights that during the 2022/23 period, there were 86 million prescriptions made for antidepressants. This figure represents an estimated patient base of 8.6 million, marking an increase of approximately 2% compared to the previous year, 2021/22. This increment translates to roughly 200,000 additional patients being identified for antidepressant prescriptions.

Increasing diagnostic capabilities, coupled with the emergence of newer psychotropic drugs, have driven a trend towards mental health polypharmacy, despite national guidelines tending to promote monotherapy [5]. While polypharmacy may improve therapeutic efficacy in certain cases, one must balance this against the potential risks, including adverse drug reactions and drug interactions. These risks should not be underestimated: one study highlighted that almost 59% of mental health inpatients had a potentially inappropriate prescription, with 33% and 12% of patients assessed as having a potentially serious or fatal inappropriate prescription, respectively [6]. Another study found the incidence of drug-related problems for patients taking antidepressants to be 16% [7]. Predictive factors of inappropriate prescriptions included polypharmacy and comorbid somatic illness, underscoring the importance of review and deprescription.

### Initiation and Failure to Examine Mental Health Polypharmacy in Primary Care

Patients with mental health conditions who are prescribed psychotropic medications are assigned a Systematised Nomenclature of Medicine Clinical Terms (SNOMED CT) code to be included in mental health lists. The coding in these lists will ensure that these patients are monitored at least annually through a series of checks, investigations, and medical examinations led by the general medical practitioner (GP).

However, due to a profound increase in the workload of primary care services and lack of funding, these mental health lists are being capped. This leads to many patients not being coded into these lists, leading to their exclusion from such routine monitoring. As such, these non-coded mental health patients are at higher risks of developing mental health polypharmacy and comorbid conditions.

**Figure 1:**
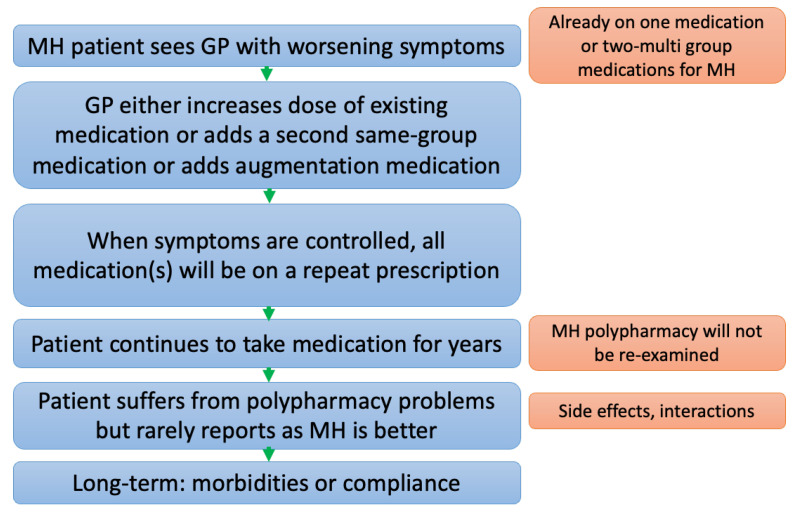
Flow diagram illustrating the steps leading to mental health polypharmacy morbidities or compliance issues in general practice.

Generally, if mental health patients experience an exacerbation of symptoms, they will consult their GP. The GP will routinely have access to each patient’s mental health records and medication history (whether initiated by a psychiatrist or GP). After an assessment, the GP may decide to increase MH medication dosage or to introduce additional medications to address this deterioration in the patient’s mental health state. Over time, and due to a lack of monitoring and medication reviews, the patient may endure the latent effects of polypharmacy, such as adverse drug reactions or drug–drug interactions. These adverse events may be under-reported due to the patient’s improved mental health status [9,10,11]. Finally, long-term morbidities or compliance issues develop. Figure 1 highlights the common pathway leading to mental health polypharmacy in the primary care setting.

**Figure 2:**
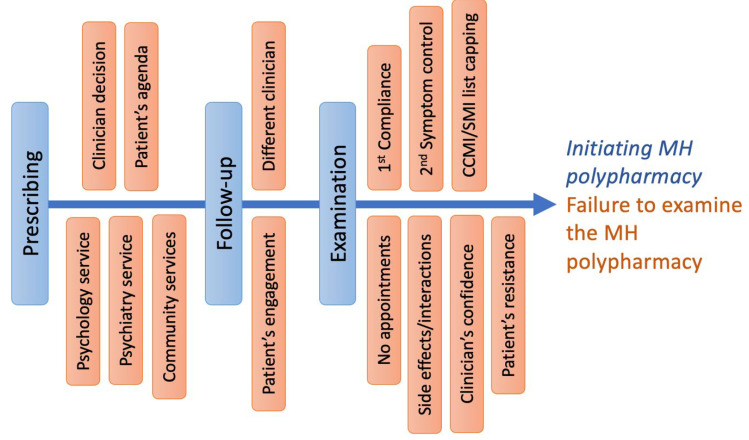
Fishbone diagram illustrating the factors that contribute to the initiation of psychiatric polypharmacy and failures to address inappropriate polypharmacy through rationalisation and deprescription. Critical decision points are also displayed, ranging from the initial prescription decision influenced by both clinician-led and patient-specific factors to the follow-up procedures that may involve multiple clinicians. This model underscores the multifaceted nature of deprescribing, highlighting areas where inappropriate prescribing may take place. Failure at these points leads to inappropriate polypharmacy.

The initiation of mental health polypharmacy by clinicians is a multifaceted decision influenced by the complexity of mental health conditions, individual patient responses to medication, comorbid conditions, patient history, evolving research and guidelines, and patient preferences. Each of these factors contributes to the careful consideration that clinicians must undertake when prescribing multiple mental health medications (Figure 2).

The inability to effectively monitor patients with mental health polypharmacy (after initiation) by GPs stems from various systemic and individual factors. Key issues include the lack of a coded mental health list for proper monitoring, inconsistent care due to patients seeing different clinicians, and clinicians’ primary focus being on symptom control and compliance over potential side effects or drug interactions. Additionally, the scarcity of GP appointments limits comprehensive medication reviews. GPs’ varying confidence in managing polypharmacy, especially for complex mental health cases, and patient resistance to medication reduction due to fears of symptom relapse also contribute to this challenge [9]. Overall, these factors lead to insufficient examinations and management of mental health polypharmacy (Figure 2).

In this prospective quality improvement project, we planned to systematically identify and address potential concerns arising from mental health polypharmacy in non-coded patients prescribed with medications from the same therapeutic group: in this case, anti-anxiety/depression medications.

## 2. Materials and Methods

An electronic search was conducted to include all mental health patients in a large-size primary care centre (North End Medical Centre) in London with a list size of nearly 20,000 patients. This resulted in the identification of 434 SNOMED CT coded-patients in mental health lists (92 CCMI: common complex mental illness and 342 SMI: serious mental illness) and 667 non-coded patients. Patients with a SNOMED CT code that allocates them into the CCMI list or SMI list are identified as needing to have annual mental health reviews, which includes annual reviews of their mental health medication. All electronic records of the non-coded patients were examined to try and identify polypharmacy as per the classification set by the NASMHPD [4].

A total of 72 non-coded patients were identified exhibiting “same-class” polypharmacy, as well as a variable number of “adjunctive” and “augmentation” polypharmacy cases. The demographics of these patients showed that 48 were male and 24 were female. The average age was 42, with the age range being 26–71 and with a standard deviation of 8. The cohort’s racial backgrounds included White (28 patients), Asian (20 patients), Black (15 patients), Mixed (6 patients), and others (3 patients).

The cohort ASA (American Society of Anesthesiologists) classification of physical status was 1 (healthy person) in 29 patients and 2 (mild systemic disease) in 43 patients. Regarding their smoking habits, 46 were non-smokers, 13 were current smokers, and 13 were ex-smokers. Regarding their alcohol consumption, 33 were non-drinkers, 25 were current drinkers, and 14 were ex-drinkers. None of the patients had a current case or previous history of chronic alcoholism or drug use. All patients were independently mobile and able to carry out their daily life activities independently. The cohort’s mental health diagnoses included mixed anxiety and depression disorder in 48 patients, post-traumatic stress disorder in 16 patients, obsessive compulsive disorder in 6 patients, and personality disorder in 2 patients.

Upon discussing the potential repercussions of polypharmacy, 68 (out of 72) patients concurred with the proposed plan for mitigating its effects. These patients were offered a mental health consultation to discuss their condition, medication, and any potential specific symptoms that could be related to medication side effects or interactions. This deprescribing prospective quality improvement project was conducted from 2021 to 2023, during which every patient was regularly reviewed face-to-face and followed up for a mean of 18 months.

Post the initial clinical review, 54 of these patients were able to attribute a portion of their presenting symptoms to their mental health polypharmacy regimen. These included headaches (18), nausea (6), dizzy spells (7), fatigue (12), abdominal pain (4), myalgia (7), arthralgia (9), back pain (14), weight gain (22), skin reactions (8), worsening anxiety (16), and sleep disturbances (19).

The “same-class” polypharmacy agents involved included: selective serotonin reuptake inhibitors (SSRIs), including Sertraline, Citalopram, Escitalopram, Paroxetine, and Vortioxetine; serotonin–norepinephrine reuptake inhibitors (SNRIs), encompassing Venlafaxine and Duloxetine; tricyclic antidepressants (TCAs), represented by Amitriptyline; and α2 adrenergic receptor antagonists, illustrated by Mirtazapine and serotonin modulators like Trazodone. On the other hand, the “adjunctive” polypharmacy agents mostly involved analgesics, anti-emetics, and medications to aide weight loss (e.g., Orlistat). Furthermore, the “augmentation” polypharmacy agents involved Propranolol, Pregabalin, Promethazine, Zopiclone, and Zolpidem.

### 2.1. Project Model

In this project, a novel model was implemented for managing mental health (MH) polypharmacy, drawing inspiration from the Plan–Do–Study–Act (PDSA) cycle. The first stage, “Idea”, is dedicated to identifying patients engaged in MH polypharmacy. The subsequent “Plan” stage involves conducting thorough patient reviews to discuss the risks associated with polypharmacy and to assess overall health status. The “Do” stage entails strategically reducing medication dosages to the lowest effective levels or discontinuing superfluous medications, concurrently optimising non-pharmacological treatment approaches. In the “Study” phase, patient monitoring is conducted to evaluate the outcomes of these interventions. Finally, the “Act” stage involves analysing the results to ascertain the efficacy and sustainability of the implemented changes. This 18-month project incorporated three such PDSA cycles, as detailed in Table 1.

This project’s primary objective was to curtail the number of prescribed mental health medications without compromising the control of mental health conditions. To this end, the count of psychotropic, adjunctive, and augmentative mental health medications for each patient was recorded at the initial consultation and subsequently at 3-month intervals, culminating in the final 18-month consultation. Concurrently, mental health control was quantitatively assessed using the Patient Health Questionnaire-9 (PHQ-9) and Generalised Anxiety Disorder Assessment-7 (GAD-7) scores at these same intervals.

Additionally, the project meticulously recorded the number of side effects patients attributed to their mental health medications, as well as the number of clinician-determined drug interactions at each time point, from the initial consultation through to the final assessment at 18 months. A comprehensive evaluation of physical characteristics was also conducted at each of these intervals, encompassing measurements of body mass index, systolic and diastolic blood pressure, heart rate, glycosylated haemoglobin (HbA1c), and cholesterol levels. This multi-faceted approach provided a holistic view of the impacts of MH polypharmacy management.

### 2.2. Ethics

This project, designed as a quality improvement project, does not necessitate formal ethics approval. Quality improvement projects are typically aimed at enhancing processes or systems within a specific organisational context, focusing on the practical implementation of the best practices rather than on experimental research. As such, they often fall outside the scope of research ethics boards. In our project, the primary objective is to improve existing procedures or outcomes within a given framework, ensuring that the activities align with standard operational policies. Thus, given its nature and objectives, this quality improvement project is exempt from the requirement of ethics approval, adhering to established guidelines and institutional policies regarding quality improvement initiatives.

Moreover, the project did not involve any interventions, manipulations, or alterations to standard patient care. All information was anonymised prior to analysis to protect the confidentiality and privacy of the individuals whose records were examined.

All activities related to data collection, storage, and analysis were conducted with the utmost care to adhere to the best practices in data management and to comply with the ethical principles surrounding research in healthcare.

## 3. Results

**Figure 3:**
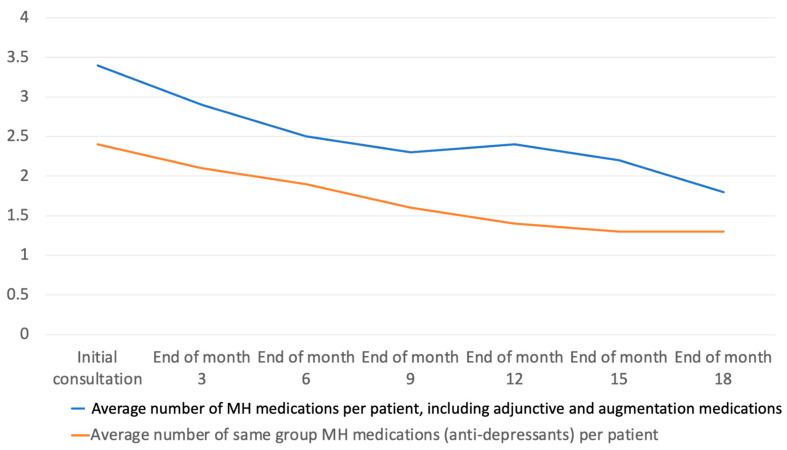
Average (mean) number of psychiatric medications per patient at specified time points during the 18-month period of the project.

In PDSA 1, a detailed analysis of medication trends over the 18-month period revealed significant findings regarding the management of mental health polypharmacy. At the outset of the project, during the initial consultation phase, the average number of psychotropic medications prescribed per patient was 2.4. This figure notably increased to an average of 3.4 medications per patient when augmentative and adjunctive medications, often used to enhance the efficacy of primary psychotropic drugs or to address side effects, were taken into account (Figure 3).

As the project progressed, a consistent downward trend was observed in the number of medications prescribed. By the end of the 18-month period, the mean number of primary psychotropic medications per patient had decreased markedly to 1.3. This reduction suggests a significant shift in the management approach, moving towards minimising the use of multiple psychotropic drugs. Furthermore, when considering augmentative and adjunctive medications, the average per patient also saw a notable decrease, falling to 1.7 medications. This decline indicates an overall successful strategy in reducing the polypharmacy burden on patients, reflecting a more streamlined and potentially safer medication regimen (Figure 3).

A notable finding was the significant reduction in the mean number of side effects and drug interactions per patient over the 18-month period. At the outset, during the initial consultation phase, patients reported an average of 1.9 side effects and drug interactions. This figure is indicative of the complex challenges faced in managing mental health polypharmacy, where multiple medications often lead to a higher propensity for adverse effects and interactions.

**Figure 4:**
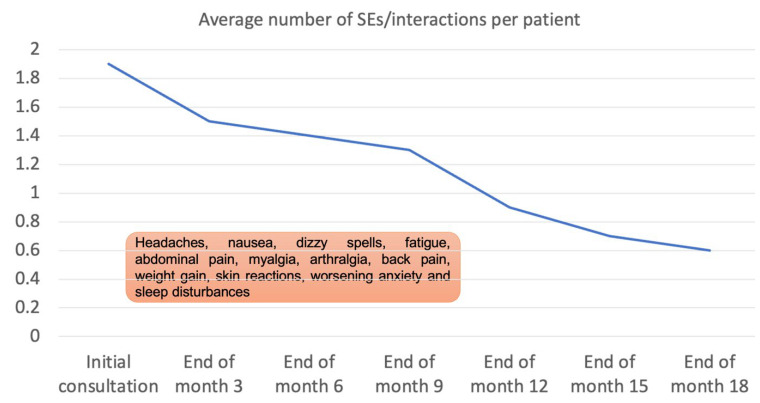
Average (mean) number of drug side effects/interactions per patient at specified time points during the 18-month period of the project.

Over the course of the project, as interventions aimed at reducing medication load and optimising treatment plans were implemented, we observed a marked decrease in these numbers. By the 18-month follow-up, the average number of side effects and drug interactions reported per patient had substantially diminished to 0.6. This reduction by more than half clearly demonstrates the effectiveness of the polypharmacy management strategies employed in this project (Figure 4).

We found that changes in medication regimens were, in most cases, sustained over time. This was a critical indicator of the success of our intervention strategies, demonstrating that patients were able to maintain their mental health stability even with reduced medication loads. We also observed that the introduction of support services played a significant role in these positive outcomes. These services provided patients with additional resources and guidance, aiding in the transition to their new medication regimens and contributing to the sustainability of these changes. The support services also offered reassurance and education, helping patients to understand and adapt to their adjusted medication plans.

**Figure 5:**
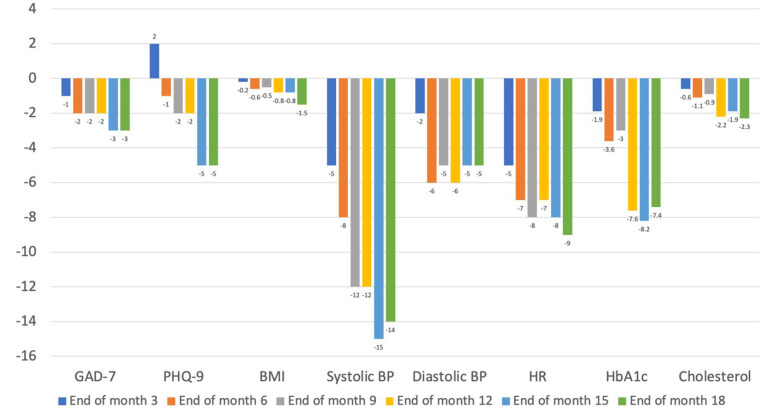
Mean changes in mental and physical health parameters at six specified time points over the 18-month project. Changes in mental health were measured using GAD-7 and PHQ-9 scores. Physical health parameters were BMI, systolic and diastolic BP, heart rate, HbA1c, and cholesterol. BMI = body mass index; BP = blood pressure; GAD-7 = Generalised Anxiety Disorder Assessment-7; HbA1c = glycosylated haemoglobin; HR = heart rate; PHQ-9 = Patient Health Questionnaire-9.

In PDSA 2, the results from patient-reported assessments indicated a notable improvement in mental health over the project period. The application of the Generalised Anxiety Disorder Assessment-7 (GAD-7) questionnaire revealed a gradual and consistent decrease in anxiety symptoms among the participants. At the 3-month follow-up, there was an average reduction of one point in GAD-7 scores compared to baseline. This improvement was more pronounced as the project progressed, with the average reduction reaching 2 points at the 6-, 9-, and 12-month follow-ups. Notably, by the 15- and 18-month follow-ups, the average GAD-7 scores decreased further, showing a reduction of 3 points below the baseline levels (Figure 5).

Conversely, the Patient Health Questionnaire-9 (PHQ-9), used to assess depressive symptoms, exhibited a transient increase initially. At the 3-month follow-up, the mean PHQ-9 score was observed to have increased by 2 points. However, this uptrend was temporary and was followed by a significant improvement in subsequent assessments. By the 6-month follow-up, the average PHQ-9 score had decreased to 1 point below the baseline. This trend of improvement continued, and by the 18-month mark, there was a substantial average reduction of 5 points from the baseline PHQ-9 scores (Figure 5).

We observed noteworthy improvements in various physical health parameters among the participants over the project’s 18-month period. These improvements were indicative of positive changes in overall health, correlating with the management of mental health polypharmacy.

A significant finding was the reduction in mean body mass index (BMI). At the end of the 18 months, the average BMI of the participants decreased by 1.5 kg/m^2^ compared to the baseline measurements. Furthermore, we noted substantial improvements in cardiovascular health indicators. There was a marked decrease in both systolic and diastolic blood pressure, with an average reduction of 14/5 mmHg at the 18-month mark relative to baseline levels. Additionally, the mean resting heart rate of the participants showed a decrease of nine beats per minute over the course of the project.

Another significant result was observed in the participants’ glycaemic control. The mean glycosylated haemoglobin (HbA1c) levels showed a decline of 7.4 points over the project period. Lastly, we also recorded a decrease in fasting cholesterol levels, with an average decline of 2.3 points.

Our follow-up assessments of the patient group revealed encouraging results. The patients demonstrated significant improvements in their mental health outcomes. These improvements were consistent across the cohort, indicating a successful application of the strategies employed in the earlier stages of the model. Additionally, follow-up evaluations showed positive changes in the physical health parameters, suggesting that the comprehensive approach to patient care, which included addressing lifestyle factors such as smoking, drinking, and social habits, had a beneficial impact.

Most notably, the non-pharmacological support provided to patients, along with the careful reviews and deprescribing of adjunctive and augmentative medications, contributed significantly to these positive outcomes. Moreover, the data indicated that these changes were sustainable over the period of the project. The continued improvements in mental health scores and physical health markers, as well as the maintenance of healthier lifestyle habits, pointed towards the long-term effectiveness of the strategies implemented.

In PDSA 3, we observed favourable outcomes across all stages of the implemented model. Education for both patients and colleagues was successfully given in educational sessions. These sessions were instrumental in enhancing the understanding of mental health polypharmacy and its management among all participants.

We developed and distributed a leaflet specifically tailored for patients, providing them with clear and accessible information about mental health polypharmacy. Additionally, we organised tutorials for clinicians, and presented the project at the AHSN Polypharmacy Network’s National Polypharmacy Programme initiative, which were aimed at equipping them with the necessary skills and knowledge to effectively manage MH polypharmacy in their practices. These educational tools were well-received and utilised, indicating a positive response to our planning efforts.

Annual checks were also proposed for patients with mental health polypharmacy. These checks would be conducted systematically and aim to provide valuable insights into the patients’ medication regimens and their overall mental health status. The consistency of these annual checks will ensure a continuous monitoring process, integral to the effective management of polypharmacy.

The feedback and data we collected demonstrated a positive impact, with patients and clinicians reporting better understanding and management of mental health medications. This phase allowed us to assess the effectiveness of our interventions in a real-world setting, yielding encouraging results.

Finally, we evaluated whether the changes implemented were manageable in a long-term context. The results showed that both patients and clinicians found the new approaches feasible and sustainable. This was evident in the continued use of educational materials, the effective integration of annual checks into routine practice, and the positive feedback regarding these changes.

## 4. Discussion

### 4.1. Challenges

In our prospective project investigating reductions in mental health medications within the context of mental health polypharmacy, several challenges emerged, particularly impacting both clinicians and patients. These challenges were integral in shaping the project’s outcomes and provided valuable insights into the complexities of managing mental health polypharmacy.

One of the primary challenges faced by clinicians was the complexity of balancing medication reduction with maintaining mental health stability. The process of tapering or discontinuing medications, especially when multiple drugs are involved, requires careful consideration of the potential withdrawal symptoms, the risk of relapse, and the interplay of different medications. This complexity often necessitated a cautious approach, sometimes leading to slower progress in medication reduction than initially anticipated.

Patients, on their part, often expressed apprehension about altering their medication regimen. This fear was rooted in concerns over the possible resurgence of mental health symptoms or the uncertainty surrounding the effects of changing their medication. Such apprehension sometimes resulted in resistance to changes in their treatment plan, posing a significant barrier to implementing polypharmacy reduction strategies.

Additionally, both clinicians and patients encountered difficulties due to the lack of a standardised protocol for reducing mental health medications in polypharmacy scenarios. The absence of clear guidelines often left clinicians reliant on their clinical judgment, which, while invaluable, could lead to variability in approaches and outcomes. This lack of standardisation also contributed to patient uncertainty, as they received different advice or treatment plans depending on each clinician’s perspective and experience.

Another challenge was the time constraints faced by clinicians, which limited their ability to conduct the thorough, in-depth consultations necessary for safely managing medication reductions. The complexity of polypharmacy cases typically requires extended consultation times to fully understand the patient’s history, current status, and to plan a tailored approach for reducing their medication. However, the reality of the clinical practice settings often meant that such extensive consultations were difficult to achieve.

Finally, monitoring the impacts of the medication changes posed its own set of challenges. Determining the effectiveness of reduced medication regimens required continuous and meticulous assessments of mental health status, often involving repeated measurements using tools like the PHQ-9 and GAD-7. This ongoing evaluation demanded significant effort and cooperation from both patients and clinicians, sometimes leading to challenges in maintaining consistent follow-up and data collection.

### 4.2. Design Challenges

It is essential to clarify our selection of the GAD-7 and PHQ-9 scales for monitoring purposes in this quality improvement project. These scales were chosen due to their well-established validity, reliability, and ease of use for assessing the severity of anxiety and depressive symptoms, respectively. Their widespread acceptance in both clinical and research settings, particularly in primary care, where quick and effective screening tools are necessary, supported their inclusion. While we recognise that these tools do not encompass the full spectrum of symptoms for OCD and PTSD, they offer valuable insight into the general mental health status of patients, particularly given the high comorbidity rates of anxiety and depressive symptoms within these and other psychiatric disorders.

The complexity and variability inherent in treating a diverse patient group with conditions such as mixed anxiety-depressive disorder, OCD, personality disorders, and PTSD were significant considerations in our project. The inclusion of such a heterogeneous group aimed to reflect the real-world complexity of mental health care, where comorbidities are common, and treatment often needs to be tailored to individual patient profiles. The rationale for combining patients with varying disorders under this deprescribing initiative was to address the overarching issue of polypharmacy, which is a concern across multiple mental health conditions. This approach allowed us to explore the potential for reducing medication load while maintaining or improving mental health stability, acknowledging the complex and overlapping pharmacological management strategies that these conditions often share.

The project’s emphasis was on a patient-centred approach, carefully weighing the potential benefits of augmentation against the risks of increased medication burden. This necessitated a flexible, case-by-case evaluation of each patient’s treatment history, symptom severity, and the presence of comorbidities. In terms of deprescribing recommendations, the project prioritised individualised care plans, guided by continuous monitoring of symptomatology through the GAD-7 and PHQ-9, as well as regular assessments of physical health and patient-reported outcomes. This methodology was designed to facilitate meaningful comparisons of treatment efficacy and safety on an individual basis, rather than attempting to directly compare outcomes across disparate diagnostic categories.

The absence of structured interviews and the use of side effects scales for comorbidity screening were noted as a limitation. However, our pragmatic approach, utilising thorough record reviews and symptom inquiries, was tailored to the primary care context, emphasising adaptability and resource efficiency. The project’s success in reducing medication numbers while improving health outcomes underscores the potential of our model. Yet, it also highlights the need for future research to establish clear guidelines for managing MH polypharmacy and understanding its long-term impacts. These insights contribute to a broader understanding of the complexities involved in MH polypharmacy management and underscore the importance of a patient-centred, adaptable approach in primary care settings.

### 4.3. Outcome

The results of our project, explored through three Plan–Do–Study–Act (PDSA) cycles over an 18-month period, provide illuminating insights into the management of mental health polypharmacy. These findings reflect significant advancements in both the strategic approach to polypharmacy and the tangible health outcomes for patients.

Initially, our analysis revealed a high baseline average of psychotropic medications, which expanded further when including augmentative and adjunctive medications. This initial data underscored the extent of polypharmacy challenges faced by patients. However, as the project progressed, a consistent reduction in medication numbers was observed, culminating in a significant decrease in both primary psychotropic and additional medications by the time of the project’s conclusion. This reduction is a testament to the effectiveness of our intervention strategies, aimed at minimising unnecessary polypharmacy while maintaining effective mental health treatment.

The reduction in the number of psychotropic and adjunct medications suggests a successful re-evaluation and optimisation of each patient’s medication plan. This could be attributed to several factors, including more effective use of non-pharmacological treatments, improved medication management, and a greater emphasis on monitoring and adjusting treatment plans as per individual patient’s needs [12,13].

The results highlight the potential for significant improvements in medication management within the context of mental health treatment. By focusing on reducing the number of medications, this project appears to have made strides in addressing the challenges of polypharmacy, which is a critical aspect of improving patient outcomes and overall treatment efficacy in mental health care.

Crucially, this project also noted a substantial decline in the average number of side effects and drug interactions per patient. This improvement directly corresponds to the reduced medication burden and highlights the benefit of our approach in enhancing patient safety and well-being.

This significant decline in adverse events not only highlights the success of the medication reduction and management approach but also underscores the potential for improving patients’ quality of life through careful and considered polypharmacy management. The reduction in side effects and drug interactions is a direct indicator of the improved safety and efficacy of the medication regimens for the patients involved [14,15].

Furthermore, this decrease in adverse effects and interactions suggests that the approach taken in this project might serve as a model for similar contexts. It underscores the importance of regular and comprehensive reviews of patient medication, especially in the context of mental health, where polypharmacy is common. The findings reinforce the need for ongoing assessments and adjustments of medication regimens to ensure optimal outcomes for patients with complex medication needs [16].

The sustained nature of the changes in the patients’ medication regimens over time was a vital indicator of the project’s success. The introduction of support services played a crucial role in this sustainability, offering patients essential resources and guidance throughout the transition period. These services not only facilitated adherence to new medication plans but also provided necessary emotional and educational support [17].

In the second PDSA cycle, patient-reported outcomes demonstrated a notable improvement in mental health, particularly in terms of anxiety symptoms. The initial increase in PHQ-9 scores, followed by a subsequent and consistent decrease, suggests a complex relationship between medication changes and mood fluctuations. However, the overall downward trend in these scores by the project’s end affirms the positive impact of our polypharmacy management strategies on mental health.

The consistent improvement in both the GAD-7 and PHQ-9 scores over time indicates the effectiveness of the interventions implemented in this project. These results underscore the potential benefits of carefully managed reductions in mental health medications in a polypharmacy context, not only in reducing anxiety symptoms, but also in improving overall depressive symptoms as the treatment progresses.

Our findings also extended to significant improvements in physical health parameters, including reductions in BMI, blood pressure, heart rate, HbA1c, and cholesterol levels. These changes not only reflect the direct effects of the adjusted medication regimens but also suggest the broader benefits of a comprehensive approach to patient care that encompasses lifestyle modifications [18].

The decline in BMI is particularly relevant, as it suggests a favourable shift in weight management, which is often a concern in patients on long-term mental health medication, especially those involving polypharmacy. The improvement in blood pressure is crucial, as hypertension is a common risk factor associated with various mental health medications. The reduction in resting heart rate can be indicative of improved cardiac function and overall cardiovascular health.

The reduction in HbA1c is also substantial, as it is a key indicator of long-term glycaemic control and can be adversely affected by some psychotropic medications. Furthermore, the reduction in fasting total cholesterol is particularly noteworthy, as elevated cholesterol levels are a known side effect of certain mental health medications and contribute to cardiovascular risk.

In the third PDSA cycle, the focus shifted to education and systemic integration of our findings. The development and distribution of patient-centred educational materials, along with clinician tutorials, were crucial in disseminating knowledge and teaching the best practices for managing mental health polypharmacy. The positive reception of these materials and the successful implementation of annual checks indicate a strong foundation for continued improvement in this area.

The project’s feedback loop provided essential insights into the real-world application and efficacy of our interventions. Both the patients and the clinicians reported a better understanding and management of mental health medications, validating the effectiveness of the strategies employed. Furthermore, the long-term feasibility and sustainability of these changes were confirmed, highlighting the potential for these practices to be integrated into routine clinical care [19,20].

### 4.4. Maintaining a Balance

The management of polypharmacy, particularly in the context of mental health, presents a complex clinical challenge. Acknowledging the inherent risks associated with polypharmacy is crucial, yet it is equally important to recognise scenarios where its use is not only necessary but also clinically justified. Such situations often arise in cases where patients present with multiple comorbidities requiring treatment from different therapeutic classes, or when monotherapy does not yield the desired clinical improvement. Consequently, deprescribing may not always be an appropriate strategy [21].

In managing these complexities, clinicians must exercise discernment, particularly in distinguishing between necessary and potentially inappropriate polypharmacy. Tools like the STOPP (Screening Tool of Older Persons’ Potentially Inappropriate Prescriptions) criteria serve as valuable resources in helping clinicians identify prescriptions that may not be suitable for specific patient profiles. The use of such tools underscores the importance of a tailored approach to medication management, considering the unique needs and health profiles of each patient [18].

Beyond the use of screening tools, continuous education for clinicians is imperative. This education should encompass updates in pharmacological research, evolving clinical guidelines, and insights into the complex interactions between various psychotropic medications. Such knowledge is essential in enabling clinicians to make informed decisions about medication regimens, particularly in the discipline of mental health, where the effects of medications can be profoundly impactful [17].

Furthermore, judicious clinical titration and strict adherence to established prescribing guidelines are key strategies for mitigating the risks associated with polypharmacy. Titration ensures that medication dosages are optimised for efficacy while minimising potential side effects. Adhering to prescribing guidelines helps maintain a standard of care that is both evidence-based and aligned with the latest clinical insights [14].

In mental health care, where polypharmacy is often a reality, understanding the balance between therapeutic necessity and risk management becomes even more critical. This balance requires rigorous clinical evaluations and continuous patient monitoring. A holistic approach that incorporates educating patients about their medication, potential side effects, and the importance of adherence is pivotal. This approach not only ensures the safety and efficacy of treatment but also empowers patients to be active participants in their care [16].

Furthermore, there is a pressing need for further research in this area. Developing clear, evidence-based guidelines on the appropriate combination of medications in mental health care is essential. Additionally, understanding the long-term effects of polypharmacy on various psychiatric populations will provide deeper insights into how these practices impact patient outcomes over time. Such research is crucial in refining polypharmacy practices, ensuring that they are both effective and safe for patients navigating the complexities of mental health disorders.

### 4.5. Future Directions

The absence of comparable studies in the literature presents both a challenge and an opportunity for future research in the field of mental health (MH) polypharmacy. This unique aspect of our project underscores the pioneering nature of our approach and highlights the critical need for further investigation into the complexities and outcomes of polypharmacy in mental health care. Future directions should focus on establishing standardised protocols for the deprescription of MH medications, taking into account the diverse needs and diagnoses of patients. Additionally, longitudinal studies are necessary to understand the long-term effects of reduced polypharmacy on mental and physical health outcomes. The development of clear guidelines for medication combinations, particularly in the context of comorbid conditions, will be essential in advancing this field. Our project lays the groundwork for these endeavours by demonstrating the feasibility and benefits of a tailored, patient-centred approach to MH polypharmacy management. The positive outcomes observed in our project, including improvements in both mental and physical health parameters, provide a promising foundation for future research to build upon, with the ultimate goal of enhancing patient care and safety in mental health care practices.

## 5. Conclusions

This project, centred on the management of mental health polypharmacy, has unveiled critical insights and demonstrated noteworthy outcomes. The project’s comprehensive approach, combining the Plan–Do–Study–Act (PDSA) model with rigorous data analyses and patient-centric interventions, has yielded significant results in both mental and physical health parameters.

Our findings indicate a marked reduction in the use of multiple psychotropic medications, which aligns with the evolving paradigm shift in mental health treatment towards minimising polypharmacy. This reduction was accompanied by a notable decline in reported side effects and drug interactions, underscoring the efficacy of this project’s medication management strategies. Such outcomes not only improve patient safety but also enhance patients’ quality of life, affirming the project’s success in addressing the complex challenge of polypharmacy.

The consistent improvement in mental health scores, as assessed by GAD-7 and PHQ-9, and the positive changes in physical health parameters like BMI, blood pressure, heart rate, HbA1c, and cholesterol levels, further validate the effectiveness of our approach. These improvements are indicative of a holistic treatment strategy that transcends mere medication management, encompassing lifestyle modifications and patient education.

The project’s educational initiatives, aimed at both patients and clinicians, played a pivotal role in enhancing understanding and management of mental health polypharmacy. The integration of such educational efforts into routine clinical practice, as evidenced by the positive feedback and sustained use of educational materials, signifies a crucial step towards long-term change in the management of mental health conditions.

Despite these positive outcomes, this project also highlighted the inherent challenges in managing mental health polypharmacy, such as the balancing act between reducing medication load and maintaining mental health stability, the variability in clinical approaches due to the absence of standardised protocols, and the complexities in patient monitoring and follow-up. These challenges underscore the need for continued efforts in optimising mental health treatment strategies.

Our project also reiterates the necessity of polypharmacy in certain clinical scenarios, particularly in patients with multiple comorbidities or those not responding to monotherapy. This necessitates a clear understanding of when and how to employ polypharmacy, balancing therapeutic efficacy with potential risks. Continuous clinician education and adherence to prescribing guidelines emerge as key elements in this balancing act.

Looking forward, the need for further research is clear. Developing specific, evidence-based guidelines for polypharmacy in mental health care is crucial. Understanding the long-term effects of polypharmacy on various mental health populations will deepen our knowledge and help refine treatment approaches, ensuring they are both effective and safe.

This quality improvement project has made significant strides in improving the management of mental health polypharmacy. By successfully reducing the medication burden while maintaining or enhancing mental and physical health outcomes, it sets a precedent for future initiatives. This comprehensive approach, encompassing medication management, patient education, and lifestyle modifications, provides a replicable model for addressing the challenges of polypharmacy in mental health care.

## Figures and Tables

**Table 1:** The three PDSA (“Plan, Do, Study, Act”) cycles conducted over 18 months undertaken as part of the quality improvement project.

Idea	Plan	Do	Study	Act
Identify same-class medication MH polypharmacy patients	Review patient to discuss polypharmacy SEs/interactions	Reduce dose of one medication to lowest possible dose or stop Review adjunctive medication	Follow-up patient evaluation to assess outcome	Check if change is sustained Offer support services
Review the same group of MH patients	Review MH, BMI, BP, HR, and smoking/drinking/social factors	Non-pharmacological support Review adjunctive/augmentation medications	Follow-up patient evaluation to assess outcome	Check if change is successful
Education: patients and colleagues	Provide leaflet for patients Provide tutorials for clinicians	Annual checks	Review response	Check if change is manageable

## Data Availability

The data presented in this study are available on request from the corresponding author.
